# Assessment of Patient Satisfaction With Inpatient Services Provided at an Acute Care Facility: A Quality Improvement Project

**DOI:** 10.7759/cureus.55511

**Published:** 2024-03-04

**Authors:** Olaniyi Fadeyi, Saviz Saghari, Ali Esmaeili, Anooshiravan Hami

**Affiliations:** 1 Internal Medicine, West Anaheim Medical Center, Anaheim, USA

**Keywords:** questionaire, descriptive study, inpatient., patient"s satisfaction, quality improvement (qi)

## Abstract

Hospitals across the United States use patient satisfaction surveys to assess the quality of inpatient and outpatient services provided to patients when they interact with the healthcare system. Results from this survey are used as input to identify weaknesses in the system with the intention of providing appropriate intervention. Here, we report the results of the quality improvement project completed in an acute healthcare facility. Patient satisfaction was evaluated based on indices established by the Hospital Consumer Assessment of Healthcare Providers and Systems (HCAHPS). Responses from 400 patients admitted into the hospital between July 2022 and June 2023 were obtained using a pre-designed questionnaire prepared by HCAHPS on behalf of Prime Healthcare Services. Indices of assessment include doctor-patient interaction, nurse-patient interaction, hospital responsiveness to patient needs, hospital environment, communication about medicine, discharge information, transition of care, overall assessment, and willingness to recommend. The best hospital performance was seen in the dissemination of discharge information, while the worst performance was noted in the transition of care and communication about medicine. Appropriate recommendations were made to improve on these weak areas.

## Introduction

The patient satisfaction survey is a form of feedback provided by patients on the quality of care received during their encounters with the healthcare system. It could provide valuable input to assess the healthcare quality and identify areas for improvement [1,2]. Therefore, it is considered a valid indicator for improving services and care delivery in healthcare organizations [3]. The patient satisfaction survey is a global trend to integrate subjective patient satisfaction responses into the evaluation of the quality of service provided in hospitals [4]. Standard questionnaires remain the most accepted evaluation tool in patient satisfaction studies [5]. Most hospitals in the United States employ the services of the Hospital Consumer Assessment of Healthcare Providers and Systems (HCAHPS) survey instrument to understand patients' perceptions of the care received while in the hospital so they can identify areas of weakness. This survey tool is very important because a low score on HCAHPS may affect hospital reimbursement from Medicare and also impact their reputation among consumers [6,7]. Our study seeks to evaluate patients' perception of the inpatient services provided at an acute care center using results retrieved from HCAHPS survey results from July 2022 to June 2023.

## Materials and methods

**Figure 1:**
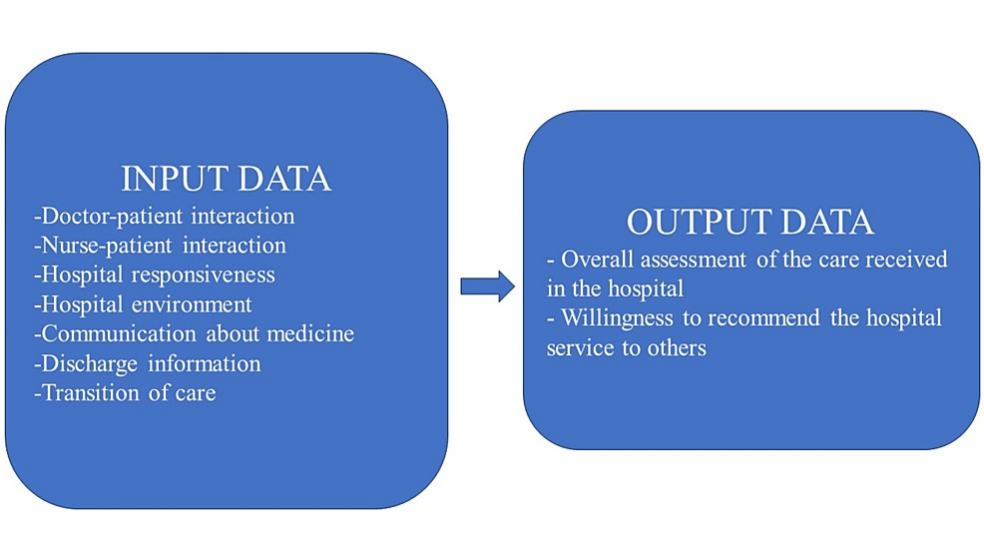
Input and output parameters

Archived data on patients' satisfaction based on the questionnaire prepared by HCAHPS was retrieved from the performance improvement department (see table in appendix). Indices of assessment in the questionnaire include nurse-patient interaction, doctor-patient interaction, hospital responsiveness, hospital environment, communication about medicine, discharge information, transition of care, willingness to recommend, and overall assessment. This study analyzed responses from 400 patients with an average age of 60-64, who were analyzed between July 2022 and June 2023. The average score for each index under review was compared with the benchmark already assigned by the Center for Medicare and Medicaid Services (see table in appendix). Also, the performance of each index across the length of the study was analyzed and presented. Further, we analyzed all the indices monthly to identify areas of weakness. Indices of assessment were categorized under two main headings: input and output data, as shown in Figure 1. The quality improvement project was approved by the ethics committee before commencement.

This is a cross-sectional study. A combination of descriptive statistics, independent sample t-test, and Spearman's rank correlation were used in the analysis.

## Results

**Figure 2:**
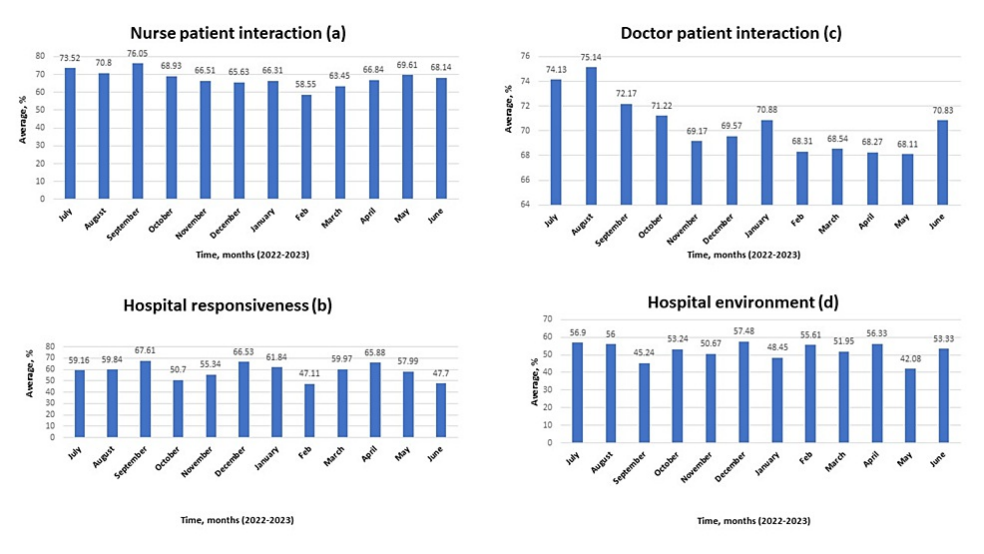
Assessment of nurse-patient interaction, hospital responsiveness, doctor-patient interaction, and hospital environment from July 2022 to June 2023 A: assessment of nurse-patient interaction; B: assessment of hospital responsiveness; C: assessment of doctor-patient interaction; D: assessment of hospital environment. Data is represented in percentages.

A review of the input data revealed the best performance with discharge information and the worst performance with transition of care (p<0.001; see table in appendix). Further analysis showed the best nurse-patient interaction and hospital responsiveness in September 2022 (Figures 2A-B), the best doctor-patient interaction in August 2022 (Figure 2C), and the best rating for the hospital environment in December 2022 (Figure 2D).

**Figure 3:**
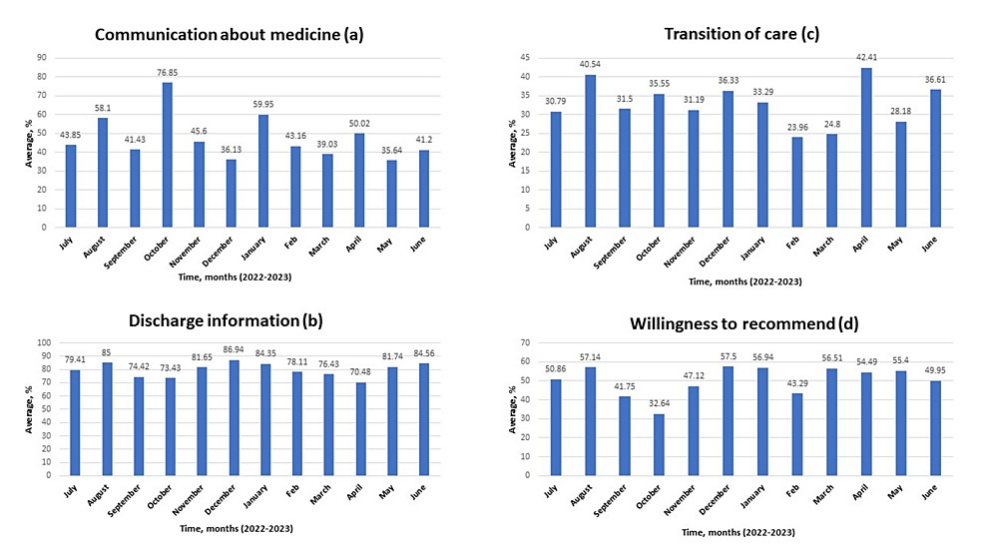
Assessment of communication about medicine, discharge information, transition of care, and willingness to recommend from July 2022 to June 2023 A: effectiveness of communication about medicine; B: effectiveness of discharge information; C: assessment of transition of care provided; D: assessment of patient's willingness to recommend the hospital Data is represented in percentages.

The best communication about medicine in October 2022 (Figure 3A), the best discharge information in December 2022 (Figure 3B), the best transition of care in April 2023 (Figure 3C), and the patient's willingness to recommend are shown in Figure 3D.

**Figure 4:**
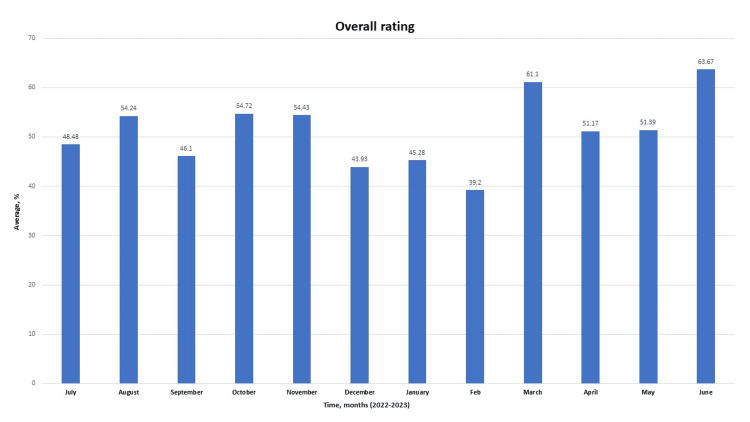
Overall rating provided by the patient over the entire study period from July 2022 to June 2023 Data is represented in percentages.

Although the best nurse-patient interaction and physician-patient interaction were seen in September 2022, a relatively low number of respondents compared to other months could have been responsible for this result. The similar pattern seen in other months with a low number of respondents would corroborate this argument. Also, regardless of the admirable physician-patient interaction seen in August 2022, it is obvious that this parameter showed a significant progressive decline across the length of the study (p<0.05; see table in appendix). While this decline may not be fully explained by the number of documented respondents, pressure on physicians to see more patients with less time spent on interaction may have been responsible for this decline. Further, it is interesting to note that regardless of the weakness in communication about medicine across the length of the study (mean of 47.58; see table in appendix), the improvement seen in this parameter in October 2022 confirmed that nurses can also augment physician efforts in this area with better nurse-patient interaction as noted in the same month. While the transition of care remains the weakest area in the acute care center under review (mean of 32.93; see table in appendix), relatively better results seen in August 2022 and April 2023 showed that improvement could be made in this regard with better physician-patient interaction, nurse-patient interaction and timely involvement of care managers in discharge plans. The monthly overall ratings expressed in percentages are shown in Figure 4.

**Figure 5:**
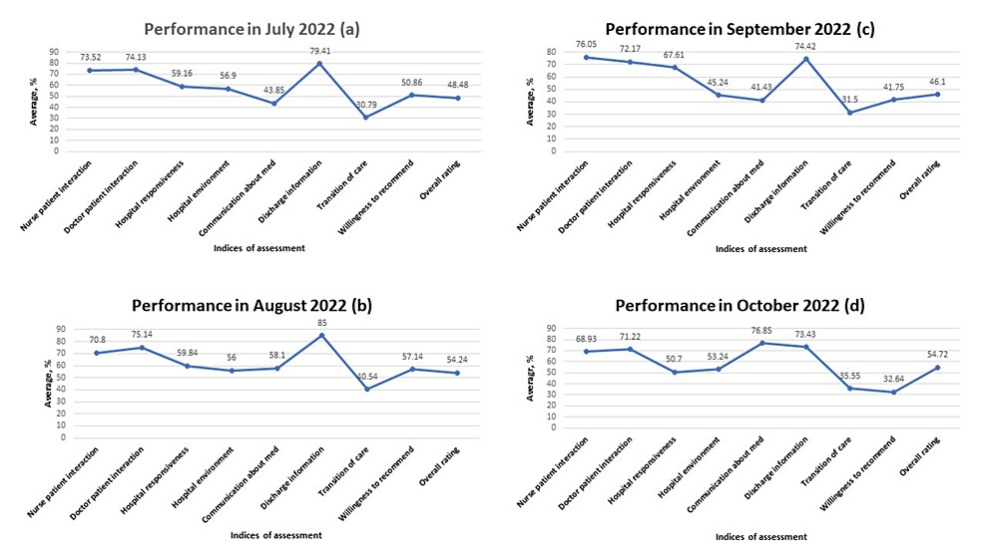
Performance across all indices of assessment from July 2022 to October 2022 A: performance across all indices of assessment in July 2022; B: performance across all indices of assessment in August 2022; C: performance across all indices of assessment in September 2022; D: performance across all indices of assessment in October 2022 Data is represented in percentages.

**Figure 6:**
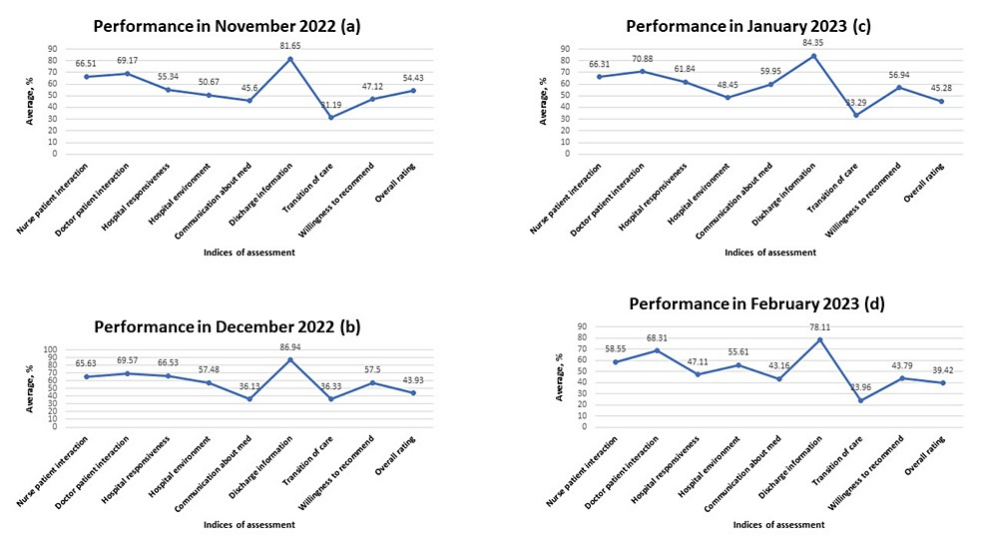
Performance across all indices of assessment from November 2022 to February 2023 A: performance across all indices of assessment in November 2022; B: performance across all indices of assessment in December 2022; C: performance across all indices of assessment in January 2023; D: performance across all indices of assessment in February 2023 Data is represented in percentages.

**Figure 7:**
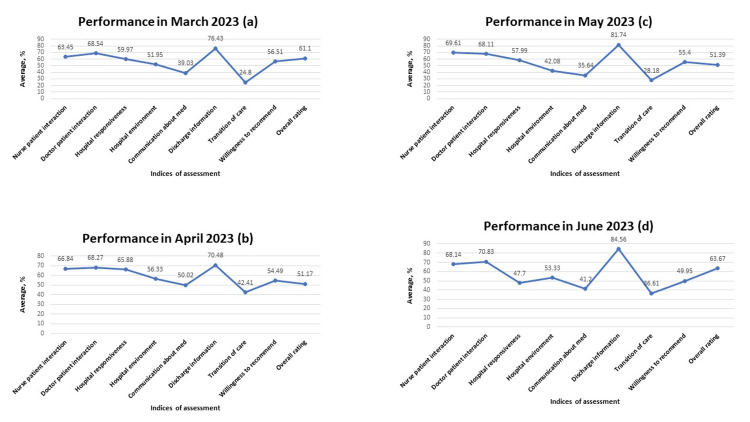
Performance across all indices of assessment between March 2023 to June 2023 A: performance across all indices of assessment in March 2023; B: performance across all indices of assessment in April 2023; C: performance across all indices of assessment in May 2023; D: performance across all indices of assessment in June 2023 Data is represented in percentages.

**Figure 8:**
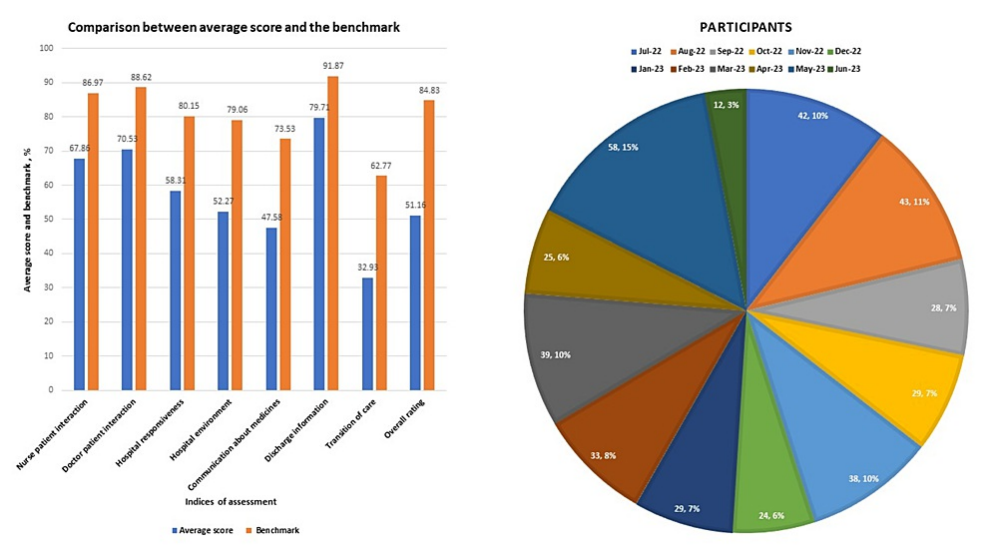
Comparison between average score with established benchmark and distribution of survey participants on a monthly basis A: Comparison between the average score of each index with the benchmark as established by CMS; B: distribution of survey participants on a monthly basis across the entire study period Data is represented in percentages. CMS - Center for Medicare and Medicaid Services

Of note, both the willingness of the patients to recommend hospital services and their overall rating are weak throughout the study (Figures 3D, 4, and 8A). Meanwhile, the admirable overall rating recorded in June 2023 is likely an outlier due to a relatively low number of respondents. Additionally, when all the parameters were compared monthly, analysis consistently revealed that performance in the dissemination of discharge information is significantly better when compared with the transition of care provided during the period under review (p<0.001; see table in appendix). Further, weakness was noted in providing effective communication about medicine (Figures 5-7).

This result is consistent with the pattern seen when the average score for each index of performance was compared with the benchmark as designated by the Center for Medicare and Medicaid Services (CMS) in Figure 8A. The distribution of the survey participants on a monthly basis is shown in Figure 8B.

Total number of respondents was 400 over 12 months. The highest survey response was received in May 2023 (58 patients), while the lowest response was in June 2023 (12 patients). A total number of respondents may have contributed to the best overall rating seen in June 2023.

## Discussion

This study aimed to examine patients' responses to questions on pre-designed questionnaire to assess the effectiveness of inpatient service provided and identify areas for improvement. Assessment of patients' satisfaction is a cost-effective way to appraise and improve the quality of health care delivery. Meanwhile, measurement of patients' satisfaction is challenging due to the overall impact of clinical and non-clinical influence on the results [8]. Nonetheless, data for this study was analyzed from different perspectives to objectively identify key areas for improvement.

For instance, the study revealed significant weakness in the transition of care. It was noted in previous studies that when patients move from the hospital to other settings, failure to implement satisfactory discharge arrangements may result in unnecessary readmissions, medication-related errors, preventable adverse events, and an increase in healthcare costs [9]. A study conducted in 2011 revealed that poorly managed care transitions resulted in complications and hospital readmissions with an associated cost of $25 to $45 billion [10]. Meanwhile, the process of transitioning patients to other care settings is a multidisciplinary one in which the hospital, healthcare providers, case managers, patients, and their families have shared responsibilities [11]. Difficulty experienced in transmitting medical information to physician practices due to the use of different electronic medical record systems is very common. It was indicated in previous studies that only 12-34% of primary care physicians have access to discharge summaries associated with patients' hospitalization at the time of follow-up [12]. In the same vein, the care transition from a non-network hospital back to the patient's medical home can be very challenging, resulting in medical complications and avoidable hospital readmissions. A situation like this is common when veterans receive inpatient services at a non-VA hospital. Consequently, different models have been developed and deployed to facilitate this process. Examples include Better Outcomes for Older Adults through Safe Transitions (BOOST), Care Transition Intervention (CTI), and the Transitional Care model (TCM). Although these models are mostly beneficial for high-risk and older populations, results have shown their effectiveness in reducing the rate of hospital readmissions and lowering overall healthcare costs [13]. A study conducted on post-hospital discharge transitional care phone calls revealed that two to three post-discharge follow-up phone calls are optimal to facilitate patients' adherence to transitional care instructions [14]. While established transition models already mentioned may not be applicable to all patients, simple post-discharge follow-up phone calls may improve patients' compliance with discharge instructions and further enhance the smooth transition of care.

Another area of weakness noted during the analysis pertains to communication about medication. Both physicians and nurses are responsible to patients in this regard. While physicians educate patients about medications in the context of the overall treatment plan, nurses have the duty to further enlighten patients on medications and administer them safely. The inability to provide appropriate education to patients about their medications and potential side effects may result in harm, readmissions, and poor outcomes [15,16]. In our analysis, weak communication about medication was noted. Careful examination of the data showed an improvement in this area in August 2022, October 2022, and January 2023 (Figures 5B, 5D, 6C). This observation confirmed that communication about medicines can be improved. Consequently, a conscious effort must be made by all care providers to carefully provide information about prescribed medications. Multiple methodology approaches encompassing the use of teach-back methods and written materials have helped to improve patients' understanding of their medications [17].

Within the present study, the hospital significantly did better in nurse-patient relationship compared with hospital responsiveness (p<0.001; see table in appendix). Hospital responsiveness is one of the major tools used in assessing the performance of the system [18]. It is defined as the ability of the healthcare system to fulfill the non-clinical desires of patients and make them comfortable [19]. It is imperative to state that a connection exists between the nurse-patient relationship and hospital responsiveness (Figures 2A-B). In the HCAHPS survey tool, most basic assistance patients request in the hospital is provided by nurses. Therefore, the nurse-patient relationship has a significant impact on how patients perceive hospital responsiveness. One study reported a negative association between nursing staff workload and patients' responsiveness [20]. Consequently, it is important to always maintain appropriate nursing staff workload to improve hospital responsiveness. 

The doctor-patient relationship in this study showed a significant progressive decline with minor variation across the 12 months under review (p<0.05; see table in appendix). While inconsistencies in the number of respondents may contribute to this observation, increasing workload may also affect the doctor-patient relationship. Physicians with increased workloads tend to spend less time with patients. Studies have shown that healthy physician-patient communication may improve health outcomes [21]. Although it is difficult to provide extensive details on physician workload because most physicians in our hospital are independent practitioners, the general pattern shows that physicians tend to have more patients during the fall and winter months. Meanwhile, good doctor-patient relationships may influence patients' satisfaction ratings and perception of the quality of care rendered by the hospital [22]. It is, therefore, necessary for physicians to take all these into consideration in the course of their clinical duty. While improvement is desired in all indices, significant improvement in the weakest areas noted in our analysis will further enhance the overall assessment of care provided and the willingness of patients to recommend the hospital service to others. Meanwhile, plans are underway to design quality improvement projects specifically targeted toward improving the weakest areas noted in this study. Such quality improvement projects will customize processes that have been successfully used in other hospitals and optimize our workflow to improve these weak areas. 

## Conclusions

Data based on responses of patients to questions on a pre-designed questionnaire prepared by HCAHPS for patients discharged from an acute care facility between July 2022 and June 2023 was analyzed in this study. Compared with the benchmark established by CMS, significant weakness was noted in the transition of care and communication about prescription medication. While different models have been used to improve the transition of care and patient-physician communication, we believe the acute care facility will improve if a designated transition coach is assigned to reinforce care transition, follow-up, and medication adherence through a post-discharge phone call. Other areas that will benefit the system include summarizing care transition while documenting information in electronic medical records (EMR) to capture succinct information on future care plan, peer-to-peer discussion between the attending physician and patient's primary care physician (PCP) on care provided to patient in the hospital and need for prompt follow-up, closing the loop on transition plan by providing information on our continuity clinic service to patients who lack PCP, providing detailed information to patients about prescription medications through written materials and use of teach-back method technique to facilitate understanding and working closely with case managers to assess facilities for patients in need of specialized care before discharge.

## Appendices

**Table 1:** HCAHPS questionnaire HCAHPS - Hospital Consumer Assessment of Healthcare Providers and Systems

Item in the questionnaire	Scale
Communication with Nurses	Never, sometimes, usually, always, yes, no
1. During hospital stay, how often did nurses treat you with courtesy and respect?	
2. During hospital stay, how often did nurses listen carefully to you?	
3. During hospital stay, how often did nurses explain things in a way you could understand?	
Communication with Doctors	
4. During hospital stay, how often did doctors treat you with courtesy and respect?	
5. During hospital stay, how often did doctors listen carefully to you?	
6. During hospital stay, how often did doctors explain things in a way you could understand?	
Responsiveness of hospital staff	
7. During hospital stay, after you pressed the call button, how often did you get help as soon as you wanted it?	
8. During hospital stay, did you need help from nurses or other hospital staff in getting to the bathroom or in using a bedpan?	
9. How often did you get help in getting to the bathroom or in using a bedpan as soon as you wanted?	
Cleanliness	
10. During hospital stay, how often were your room and bathroom kept clean?	
Quietness	
11. During hospital stay, how often was the area around your room quiet at night?	
Communication about medicine	
12. During hospital stay, were you given any medicine that you had not taken before? (Yes/No)	
13. Before giving you any new medicine, how often did the hospital staff tell you what the medicine was for?	
14. Before giving you any new medicine, how often did the hospital staff describe possible side effects in a way you could understand?	
Discharge Information	
15. During this hospital stay, did doctors, nurses or other hospital staff talk with you about whether you would have the help you needed when you left the hospital? (Yes/No)	
16. During this hospital stay, did you get information in writing about what symptoms or health problems to look out for after you left the hospital? (Yes/No)	
Transition of care	Strongly disagree, disagree, agree, strongly agree
17. During this hospital stay, staff took my preferences and those of my family or caregiver into account in deciding what my health care needs would be when I left	
18. When I left the hospital, I had a good understanding of the things I was responsible for in managing my health	
19. When I left the hospital, I clearly understood the purpose for taking each of my medications	
Overall Rating of the Hospital	0 - 10
20. We want to know your overall rating of your stay at [Hospital name]. This is the stay that ended around [discharge date]. Please do not include any other hospital stay in your answer	
Willingness to Recommend	Definitely no, probably no, probably yes, definitely yes
21. Would you recommend this hospital to your friends and family?	

**Table 2:** Benchmark established by CMS to assess patients' satisfaction CMS - Center for Medicare and Medicaid Services

Indices of assessment	Benchmark (%)
Nurse-patient interaction	86.97
Doctor-patient interaction	88.62
Hospital responsiveness	80.15
Hospital environment	79.06
Communication about medicine	73.53
Discharge information	91.87
Transition of care	62.77
Overall rating	84.83

**Table 3:** Descriptive statistics for quality indices

Variables	Statistics		Value
Nurse-patient interaction (%)	Mean		67.86
	95% confidence interval for mean	Lower bound	64.97
		Upper bound	70.76
	Median		67.49
	Standard deviation		4.56
	Minimum		58.55
	Maximum		76.05
	Range		17.50
Doctor-patient interaction (%)	Mean		70.53
	95% confidence interval for mean	Lower bound	69.04
		Upper bound	72.01
	Median		70.20
	Standard deviation		2.340
	Minimum		68.11
	Maximum		75.14
	Range		7.03
Hospital responsiveness (%)	Mean		58.31
	95% confidence interval for mean	Lower bound	53.88
		Upper bound	62.73
	Median		59.50
	Standard deviation		6.971
	Minimum		47.11
	Maximum		67.61
	Range		20.50
Hospital environment (%)	Mean		52.27
	95% confidence interval for mean	Lower bound	49.17
		Upper bound	55.38
	Median		53.29
	Standard deviation		4.887
	Minimum		42.08
	Maximum		57.48
	Range		15.40
Communication about medicine (%)	Mean		47.58
	95% confidence interval for mean	Lower bound	39.94
		Upper bound	55.22
	Median		43.51
	Standard deviation		12.02
	Minimum		35.64
	Maximum		76.85
	Range		41.21
Discharge information (%)	Mean		79.71
	95% confidence interval for mean	Lower bound	76.40
		Upper bound	83.02
	Median		80.53
	Standard deviation		5.22
	Minimum		70.48
	Maximum		86.94
	Range		16.46
Transition of care (%)	Mean		32.93
	95% confidence interval for mean	Lower bound	29.30
		Upper bound	36.56
	Median		32.39
	Standard deviation		5.71
	Minimum		23.96
	Maximum		42.41
	Range		18.45

**Table 4:** Independent sample t-test between discharge information and transition of care

Quality index	N	Mean	Standard deviation	Standard error mean	p-value
Discharge Information (%)	12	79.71	5.22	1.50	<0.001
Transition of care (%)	12	32.93	5.71	1.65	

**Table 5:** Independent sample t-test between nurse-patient interaction and hospital responsiveness

Quality index	N	Mean	Standard deviation	Standard error mean	p-value
Nurse-patient interaction (%)	12	67.86	4.56	1.32	<0.001
Hospital responsiveness (%)	12	58.31	6.97	2.01	

**Table 6:** Correlation between time in months and doctor-patient interaction

Correlation		Doctor-patient interaction (%)
Time in months	Correlation coefficient	-0.797^**^
	Significance (2-tailed)	0.002
	N	12
